# Self –reported knowledge and understanding of added sugars by consumers in Ghana

**DOI:** 10.1016/j.heliyon.2024.e31243

**Published:** 2024-05-14

**Authors:** Remember Roger Adjei, Amanda Sarfo Boateng, James Owusu-Kwarteng

**Affiliations:** a Department of Horticulture and Crop Production, School of Agriculture and Technology, University of Energy and Natural Resources, Dormaa Ahenkro Campus, P. O. Box 214, Sunyani, Ghana; b Department of Food Science and Technology, School of Agriculture and Technology, University of Energy and Natural Resources, Dormaa Ahenkro Campus, P. O. Box 214, Sunyani, Ghana

**Keywords:** Added sugars, Food labels, Consumers, Sweeteners, University, Natural sugars

## Abstract

The consumption of added sugars has been a major concern among consumers and researchers around the world. Some of these added sugars pose health threats such as obesity, and liver diseases to consumers. Therefore, consumers' understanding and knowledge of added sugars is important in regulating the intake of food items that contain different types and levels of added sugar. In this study, the knowledge and understanding of staff (consumers) of the University of Energy and Natural Resources, Ghana, was assessed through survey The results showed that about 38.5 % of consumers always read food labels whereas 3.1 % never read the labels of food they purchased. However, only about 20 % of consumers considered added sugars as most important information on food labels while most (about 38 %) were concerned about the calorie level in food items purchased. Based on the consumer's knowledge of sugars and sweeteners, there was a high level of disparity in classifying sugars in food as sugars and sweeteners. In addition, most consumers reported that they would adversely avoid food items containing lactose, isoglucose, and saccharin. The awareness of the consumers to the WHO recommendation for sugar reduction, the gender (P = 0.278), age (P = 0.959), level of education (P = 0.888), and staff category (P = 0.944) did not influence their decisions on purchasing food items with added sugars Most consumers were interested in issues of food and nutrition. Therefore, it is recommended that staff are taken through aspects of food nutrition as well as the consumption of added sugar towards the recommended levels.

## 1 Introduction

The term “sugars” includes intrinsic sugars, which are those incorporated within the structure of intact fruit and vegetables; sugars from milk (lactose and galactose); and free sugars, which are monosaccharides and disaccharides added to foods and beverages by the manufacturer, cook or consumer, and sugars naturally present in honey, syrups, fruit juices and fruit juice concentrates (WHO, 2015) [24]. According to Gillespie et al. (2023) [7], added sugars include those that are artificially added to foods and beverages. However, all added sugars are also free sugars, which are naturally occurring sugars present in dairy foods, (fresh, cooked, or dried) fruit, and vegetables. The major difference between added sugars and free sugars is that added sugars include all naturally occurring sugars in non-intact (i.e., juiced or pureed) fruit and vegetables (Mela and Woolner, 2018) [15].

Free sugars contribute to the overall energy density of diets and may promote a positive energy balance (Johnson et al., 2009; Elia and Cummings, 2007) [6,12]. Sustaining energy balance is critical to maintaining a healthy body weight and ensuring optimal nutrient intake (Hill et al., 2013) [25]. There is increasing concern that the intake of free sugars, particularly in the form of sugar-sweetened beverages increases overall energy intake and may reduce the intake of foods containing more nutritionally adequate calories, leading to an unhealthy diet, weight gain, and increased risk of non-communicable diseases (NCDs) (Hauner et al., 2012; Malik & Hu, 2012; Vartanian et al., 2007) [9,14,21]. Therefore, overconsumption of foods that are high in added sugars may replace other more nutrient-dense foods, resulting in nutrient deficiencies or overconsumption of calories (Hess et al., 2012) [10]. Consumers believe sugar is higher in calories than other macronutrients, including carbohydrates (Patterson et al., 2012) [16]. The World Health Organization recommendations are for <10 % of total energy from “free sugars”, which include both added sugars and all sugars present in 100 % fruit juice (WHO, 2015) [24]. Despite similar recommendations, the difference between “free sugars” and “added sugars” may lead to significantly different implications in specific age groups who regularly consume 100 % fruit juice.

For consumers to have a fair idea of food products, there is the need to provide labels (Cheftel, 2005) [2] and to also achieve good healthy eating, nutrition label is an important factor (Cowburn and Stockley, 2015) [3] because the point of purchase it serves as a source of information which can influence consumers purchasing power (Brownell and Koplan, 2011) [1]. Furthermore, labelling of food products communicates to buyers about the quantity and specific products to avoid thereby protecting the buyer from overuse or underuse of the products. Therefore, this research aimed to evaluate consumers' self-reported knowledge and understanding of different forms of sugars (natural, added, and free sugars) presented on food labels as well as consumers’ awareness of the WHO guidelines in relation to sugar intake.

## 2 Methodology

### 2.1 Study area and data collection

The present study was conducted at the University of Energy and Natural Resources (UENR), Sunyani-Ghana, in 2019. UENR is a public-funded university established by an Act of Parliament (Act 801).

We employed the use of the mixed methods research design (quantitative and qualitative research design) in the study and the convenience sampling technique was used in sampling the members of the university community (i.e., Junior Staff, Senior Staff, and Senior Members both teaching and non-teaching staff categories) based on their accessibility, willingness, or availability to participate in the study (Dörnyei, 2007) [5]. A total of ninety-six (96) staff willingly participated in the survey. A semi-structured questionnaire was adopted from the study of Tierney et al. (2017) [20] and was modified to suit the purpose of the present study. The questionnaire underwent a pre-testing phase among researchers and some staff members to identify and rectify any potential errors or biases. Following this refinement process, the questionnaire was structured to systematically gather pertinent information from the study participants. Additionally, a screening question was included to ensure informed consent from respondents during the administration of the questionnaire.

Section A of the questionnaire included questions on the consumer's knowledge and understanding of added sugars. The frequency of looking at nutritional labels when purchasing food (1 = Always – 4 = Never). Also, the items on the labels that consumers (i) looked at during the purchasing of food, (ii) interested them most, and (iii) most important to watch to stay healthy (1 = Calories, 2 = Total Fat, 3 = Saturated Fat, 4 = *Trans*-fat, 5 = Total carbohydrate, 6 = Total Sugar, 7 = Salt, 8 = Other [*Please specify*]).

Further, the knowledge of staff in the categorization of sugar sources was assessed by providing the list of 16 ingredients for classification into natural sugar, added sugar, or artificial sweeteners. Consumers' knowledge of the WHO recommendation and guidelines of 2015 in recommending sugar was assessed. Section B solicited information on the demographic characteristics of the respondents (age, gender, highest level of education, category of staff etc.). A detailed description of the questionnaire used for the present study has been added as supplementary material.

### 2.2 Methods of data analysis

The filled-out paper questionnaire was coded and entered in Microsoft Excel, 2016. All statistical analysis was performed with IBM Statistical Package for Social Science for Windows (version 26.0) (IBM Corp, 2019) [11]. Demographic characteristics of the participants were assessed using descriptive statistics in the form of frequencies and percentages. Consumers' knowledge and understanding of free sugars were also analysed using descriptive statistics (frequencies and percentages). Cross-tabulation was used to analyse consumers’ awareness of WHO recommendations for sugar reduction.

## 3 Results

### 3.1 Respondent's profile

The majority of the respondents were male (58.3 %) and females were 41.7 %. With regards to the age group of the staff, 25–34 years old constitute most of the respondents representing 57.3 %, followed by 35–44 years old with a percentage of 18.8 %. However, 7.3 % of respondents preferred not to disclose their age. At the time of this survey, 49 % of the respondents had a postgraduate degree, 42.7 % had a bachelor's degree, while only a few (7.3 %) of respondents preferred not to disclose their level of education. Furthermore, a greater number of the respondents (59.4 %) were senior staff, 25 % were senior academic staff while the junior staff category constituted 8.3 %. Additionally, 69.8 % of respondents had children under 18 years of age in their households (Table 1).

**Table 1:** Socio-demographic profile of respondents.

Characteristics	Frequency (n = 96)	Percentage (%)
Gender		
Male	56	58.3
Female	40	41.7
***Age (Years)***		
18–24	3	3.1
25–34	55	57.3
35–44	18	18.8
45–54	6	6.3
55–64	5	5.2
65–74	1	1.0
Prefer not to say	7	7.3
***Level of education***		
High School	1	1.0
Bachelor's degree	41	42.7
Postgraduate	47	49.0
Prefer not to say	7	7.3
***Staff category***		
Senior Member Teaching	24	25.0
Senior Member Non-Teaching	7	7.3
Senior Staff	57	59.4
Junior Staff	8	8.3
***Children under 18 in household***		
Yes	67	69.8
No	29	30.2

### 3.2 Use of nutrition information

**Fig. 1:**
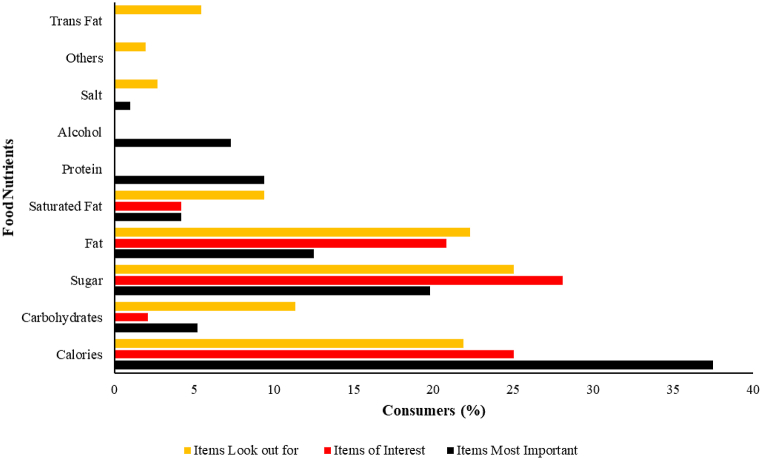
Nutrition panel items of concern to consumers.

In general, 47.9 % of respondents reported that they “sometimes” read food labels and 38.5 % “always” read food labels when buying packaged foods. However, 10.4% of the respondents “hardly ever” read food labels whereas 3.1 % “never” considered reading food labels (Table 2). The use of labels among respondents showed no significant differences regarding gender (p = 0.340), age (p = 0.191), education level (p = 0.326), and the category of staff (p = 0.407). Participants were questioned on which nutrition items on the food label interested them during the purchase of food. About 28.1 % of the respondents representing the majority choose Sugar followed by Calories (25 %) and fat (20.8 %) as the items in particular that are of interest to them. With regards to items on the labels that the staff does not look out for include calories (21.9 %), sugar (25.0 %), fat (22.3 %), and carbohydrates (11.3 %). Nonetheless, the items most important to the respondents were calories (37.5 %), sugar (19.8 %) and fat (12.5 %) (Fig. 1).

**Table 2:** Frequency of staff looking at food labels.

Frequency	n = 96	%
Always	37	38.5
Sometimes	46	47.9
Hardly Ever	10	10.4
Never	3	3.1

### 3.3 Consumers’ knowledge of sugars and sweeteners on food labels

Respondents were asked to classify the commonly used food ingredients as natural sugars, added/free sugars, or artificial sweeteners (Table 3). Most respondents could not correctly classify the food ingredients as natural sugars, added or free sugars, or artificial sweeteners. Glucose was incorrectly classified by most of the respondents (66.7 %), while only 18.8 % classified glucose correctly, and 14.5 % of the respondents did not know what category glucose belongs to. Also, sucrose was incorrectly classified (51 %), whereas some of the respondents (19.8 %) classified sucrose correctly as added sugars and 27.1 % had no idea about the correct category of sucrose. Progressively, only 10.4 % classified fructose correctly, and 13.5 % classified maltose correctly as added sugars. However, the following food ingredients; honey (6.3 %), agave nectar (9.4 %), molasses (15.6 %), fruit juice (18.8 %), corn syrup (16.7 %), inverted sugars (14.6 %), and isoglucose (19.4 %) were correctly classified by consumers as added sugars. With regards to the classification of the artificial sweeteners, 18.8 % and 23.9 % of the respondents correctly classified saccharin and aspartame as artificial sweeteners, respectively.

**Table 3:** Respondent's ability to classify dietary sugars and sweeteners.

Classifications	Correct	Wrong	Don't Know
	n (%)	n (%)	n (%)
Glucose	18 (18.8)	64 (66.7)	14 (14.5)
Sucrose	19 (19.8)	51 (53.1)	26 (27.1)
Saccharin	18 (18.8)	25 (26)	53 (55.2)
Fructose	10 (10.4)	54 (56.3)	32 (33.3)
Maltose	13 (13.5)	48 (50)	35 (36.5)
Honey	6 (6.3)	82 (85.4)	8 (8.3)
Agave nectar	9 (9.4)	49 (51.0)	38 (39.6)
Molasses	15 (15.6)	27 (27.1)	54 (56.3)
Fruit juice	18 (18.8)	69 (71.9)	9 (9.4)
Corn syrup	16 (16.7)	49 (51.0)	21 (32.3)
Aspartame	23 (23.9)	8 (8.3)	65 (67.7)
Invert sugar	14 (14.6)	30 (31.3)	52 (54.2)
Isoglucose	9 (9.4)	35 (36.5)	52 (54.2)
Lactose	32 (33.3)	21 (21.9)	43 (44.8)
Sugars in fresh fruits and vegetables	52 (54.2)	7 (7.3)	37 (38.5)

Most of the respondents (33.3 %) correctly classified sugars present in milk (lactose) as natural sugars and 44.8 % did not know the class of lactose. Additionally, 21.9 % of respondents wrongly classified sugars present in milk as added or free sugar. Sugars present in fresh fruits and vegetables were correctly classified by the respondents as natural sugar, and 7.3 % classified sugars in fresh fruit and vegetables as added sugars.

**Fig. 2:**
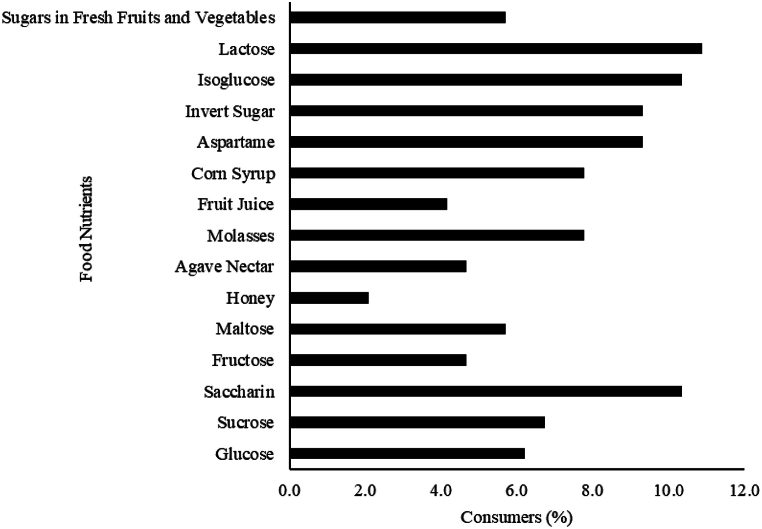
Sugars that consumers would avoid.

In terms of which sugars consumers will actively avoid, the majority (10.9 %) stated they would avoid lactose, followed by isoglucose and saccharin (10.4 %) whereas only a few of the consumers (2.1 %) stated that they would avoid honey (Fig. 2). Other food item that most consumers would avoid is invert sugar (9.3 %), aspartame (9.3 %), corn syrup (7.8 %), and molasses (7.8 %).

### 3.4 Consumers’ awareness of WHO recommendation for sugar reduction

The awareness of the consumers to the recommendation of WHO on sugar reduction was assessed. The majority (62.5 %) of consumers reported that they were not aware of the World Health Organization (WHO) recommendations for sugar reduction, whereas 37.5 % were aware of the WHO recommendation for the reduction of added sugars to 5 % daily intake for additional health benefits. There was no significant difference (*p > 0.05*) for the awareness of the WHO recommendations with regards to gender of respondents (p = 0.278), age (p = 0.959), level of education (p = 0.888) and staff category (p = 0.944) Table 4).

**Table 4:** Consumers’ awareness of WHO recommendation for sugar reduction.

	Aware (%)	Not Aware (%)	*P value*
Awareness of WHO recommendations	37.5	62.5	
Gender			
Male	42.9	57.2	0.278
Female	30.0	70.0	
**Age (Years)**			
18–24	0.0	1.8 %	0.959
25–34	36.4	63.6	
35–44	50.0	50.0	
45–54	50.0	50.0	
55–64	40.0	60.0	
65–74	0	100	
Prefer not to say	28.6	71.4	
**Level of Education**			
College	0.0	100	0.888
Degree	34.1	65.8	
Postgraduate	40.0	59.6	
Prefer not to say	42.9	57.1	
**Staff Category**			
Senior Member Teaching	41.7	58.3	0.944
Senior Member Non-Teaching	28.6	71.4	
Senior Staff	38.6	61.4	
Junior Staff	25.0	75.0	

**Fig. 3:**
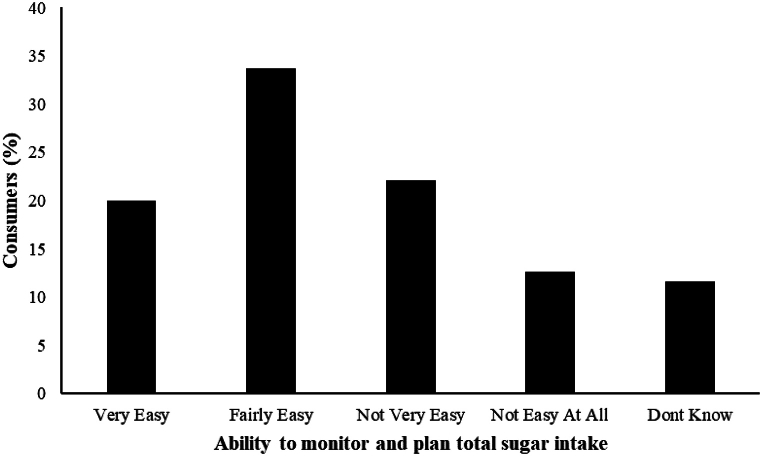
Consumers perceived ability to monitor and plan their total sugar intake based on WHO guidelines.

**Fig. 4:**
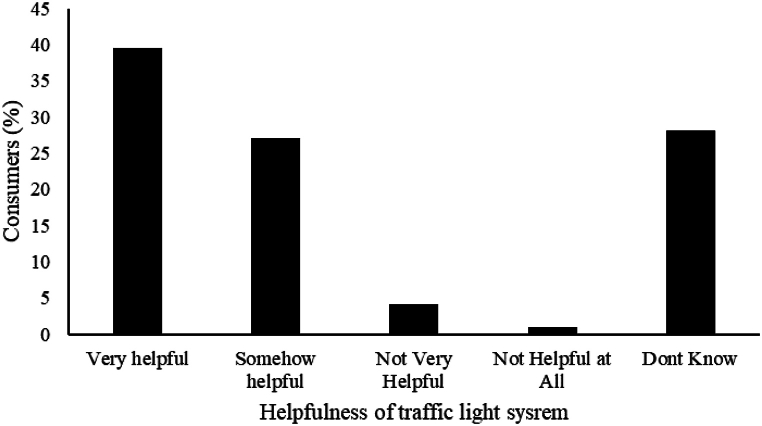
Helpfulness of traffic light system of food labelling for consumers.

Based on WHO guidelines on the current labelling, respondents were asked to indicate how easy it would be to monitor and plan for their intake of total sugars. The consumers indicated that it would be fairly easy (33.7 %), Not very easy (22.1 %), Very Easy (20 %), and Not Easy at all (12.6 %) in monitoring their intake of total sugars (Fig. 3). The traffic light system of labelling food was ranked as very helpful by the majority of the consumers (39.5 %) interviewed. Only a few (1 %) of the respondents rated the traffic light system as not helpful at all (Fig. 4).

Based on the staff's interest in food and nutrition, most of the participants (46.9 %) indicated that they are very much interested whereas only a few of the staff have no idea of their interest in issues of food and nutrition (Table 5). In the respondent's responses to the open-ended question which questioned consumers' approach to managing their sugar intake with regards to reducing consumption, most of the respondents constituting more than half of the total respondents alleged that they would either reduce or avoid the intake of sugars while a little portion of the respondents stated that they would avoid canned foods and packed fruit juices. However, the most important aid in understanding the sugar content is to look at the label of the foodstuff that is been purchased.

**Table 5:** Consumers self-reported level of interest in food and nutrition issues.

Variables	n = 96	%
Very Interested	45	46.9
Interested	42	43.8
Not Very Interested	8	8.3
Don't Know	1	1.0

## 4 Discussion

Our study focused on consumers' knowledge and understanding of added sugar among university staff at the University of Energy and Natural Resources, Sunyani-Ghana. We found out that most of the university staff who represented the consumers interviewed were of the youthful age group of 25–34 years old and all the respondents have had higher education which suggested that the respondents had an appreciable knowledge and understanding of food nutrition. The present study observed that a greater part of the respondents critically looks at food labels often to know the kind of food ingredients in the food products they purchase. This could be attributed to their level of education. A similar finding was confirmed from the studies of Loureiro et al. (2006) [13] where they explained that highly educated consumers are more likely to read scientific, academic articles or prints and are more likely to be exposed to health and nutrition-related news sources. These, in turn, increase respondents' awareness of diet and health-related issues. Similarly, Prada et al. (2020) [17] reported a high level of education with adequate knowledge of nutritional guidelines among the respondents in their study in Portugal. Nevertheless, in the study of Darkwa (2014) [4], they reported that 80 % of the respondents were interested in reading nutrition information on the labels of packaged foods before buying and 45 % of the respondents had attained a higher level of education. Washi's (2012) [22] study also observed that many participants (89.5 %) showed a general awareness of food label information including nutrition label information and 69.5 % of the participants were graduates.

Further, most of the consumers were interested in looking at the sugar, calories, and fat content in their foods. About 28 % of the consumers were particular about the sugar content in their food. These could suggest that the consumers in our study were particularly careful with the intake of sugar, calories, and fat in whatever they bought from the market. Similar findings were reported by Grunert et al. (2010) [8] and Tierney et al. (2017) [20]. The participants in our study also reported that the most important items to watch on their food labels are calories followed by sugars and fats. Some research has associated excessive consumption of these dietary nutrients to certain diseases which include hypertension, diabetes, cancer, obesity, and a whole lot (Whitney & Rolfes, 1993; Sienkiewicz & Sizer, 2011) [19,23].

Consumers’ knowledge of sugars and sweetness on food labels was assessed. The majority of the participants were not able to classify the added sugars and sweeteners correctly. This suggests that the consumers do not correctly understand the difference between natural sugars, added sugars, and added sweeteners. However, few of the participants have an idea about the classification of sugars and sweeteners. Interestingly, most of the respondents also did not know what category to classify the dietary sugars and sweeteners. Our present study also revealed that some of the natural sugars were wrongly classified as added sugars by respondents. Similarly, Tierney et al. (2017) [20] suggested that educating consumers on how to identify added sugars in their foods may be important. The consumers were particular that, they would actively avoid lactose followed by Isoglucose and saccharin actively in their foods. However, few of the respondents indicated that they would avoid Honey.

Our study also focused on consumers’ awareness of WHO recommendations for sugar reduction. The World Health Organization (2015) [24] gave a strong recommendation that there should be a reduced intake of free sugars throughout the life course of both adults and children, to a reduced level of less than 10 % of total energy intake and suggests a further reduction of the intake of free sugars to below 5 % of total energy intake. Based on our findings with regard to the level of education, the majority of the respondents were not aware of the WHO recommendations. This suggests that despite the high level of education among the respondents, they might not have gotten access to the WHO guideline for sugar intake for adults and children. Furthermore, the participants feel it would be fairly easy to monitor their sugar intake with the WHO guidelines but some of the respondents feel it would not be an easy task to undertake. This shows that the respondents were willing to adapt and embrace the WHO guidelines to help them in the regulation of their daily intake of sugars.

The consumers indicated that the traffic light system of food labelling would be very helpful in checking their food intake. The traffic light system has been widely used among researchers due to its simplicity, clarity, and ease of comprehension (Kunz et al., 2020; Balcombe et al., 2010; Sonnenberg et al., 2013) [[26], [27], [28]] and helps consumers assess the healthiness of food products conveniently. It has been found to encourage healthy eating among consumers as well as promote awareness of healthy choices during shopping (Balcombe et al., 2010; Sonnenberg et al., 2013) [27,28]. In our study, most of the consumers have no idea about the traffic system. Based on the open-ended questions, respondents highlighted that they would look at the food label information to regulate their sugar intake and avoid packed food. Our results suggested that food label information is a very crucial way of getting important good information about the food they are purchasing and is an important source of new knowledge that can aid consumers when doing some purchasing. One of the key components on the food label is the expiry date and Sabbe et al. (2009) [18] found that the expiry date is commonly used by consumers as an illustration of freshness, shelf life, and food safety across a range of foods. The Food and Agriculture Organization of the United Nations working towards zero hunger highlighted some of the benefits of food labelling as it helps in understanding what is in the food, ensures food safety through storage and cooking instruction, helps prevent the buying of counterfeit products, prevents harmful reactions to food and provides date marking to reduce food wastage.

## 5 Conclusion and recommendation

The study aimed to assess consumers' self-reported knowledge and understanding of added sugars among the staff of the University of Energy and Natural Resources, Sunyani, Ghana. Our present study adds to the existing knowledge of consumers’ knowledge and understanding of added sugars among university staff in Ghana. To the best of our knowledge, this is the first of such study conducted among university staff in Ghana and presents the importance of understanding the types and importance of added sugars in their food. The results showed that most of the staff who represented the consumers had little knowledge about artificial sugars and sweeteners. It was also found that most of the consumers were not aware of the traffic light system for food label information. In addition, consumers have been exposed to the use of traffic light systems for food label information and the need to adopt them in their quest for purchasing or selecting food items. Based on the results obtained we recommend that consumers (staff) and the general public be educated on artificial sugars and sweeteners and encouraged to read the WHO recommendations on food labelling and Intake. We also recommend that consumers are given adequate education on their food intake and the information on food labels for a better understanding of using the traffic light system for healthiness.

## 6 Limitation of the study

The understanding of added sugars may vary among staff members based on cultural dietary practices, making it challenging to generalize findings across diverse populations. Also, the study was conducted among the staff of the University of Energy and Natural Resources, Sunyani-Ghana, so therefore, due to the staff population during the time of the study, the findings may not accurately reflect the knowledge and understanding of added sugars among all staff members. In addition, staff members may not fully understand the concept of added sugars or may confuse it with naturally occurring sugars in foods, leading to inaccurate self-reports as reported in the study.

## Ethics statement

Review and approval by an ethics committee were not necessary for this study but participants were informed that the data collected would be exclusively used for academic purposes, thereby ensuring the confidentiality of their identities. Furthermore, it was made clear that individuals selected for the present survey retained the prerogative to opt-out at any point during the interview process or decline to respond to specific questions should they find them discomforting.

## Data availability statement

Data will be made available upon reasonable request from the corresponding author.

## Funding

Not applicable.

## CRediT authorship contribution statement

**Remember Roger Adjei:** Writing – original draft, Visualization, Software, Methodology, Investigation, Formal analysis. **Amanda Sarfo Boateng:** Writing – review & editing, Resources, Methodology, Investigation, Data curation. **James Owusu-Kwarteng:** Writing – review & editing, Supervision, Resources, Conceptualization.

## Declaration of competing interest

The authors declare that they have no known competing financial interests or personal relationships that could have appeared to influence the work reported in this paper.
