# Supplementary material for: Self –reported knowledge and understanding of added sugars by consumers in Ghana
Source: Heliyon. 2024 May 14;10(10):e31243. doi: 10.1016/j.heliyon.2024.e31243 (PMC11128992; doi:10.1016/j.heliyon.2024.e31243)
Supplement: Multimedia component 1 [file mmc1.docx]

Consumers’ Self-reported Knowledge and Understanding of Added Sugars

**Questionnaire**

We are conducting a survey on labelling information of foods and would be very grateful if you could take some time to answer these questions. We estimate that it will take around 5-6 min.

Informed Questions:

Respondents must answer ‘yes’ to all screening questions before progressing to Q1.

1. I confirm that I have been given and have read and understood the information sheet for the above study and have asked and received answers to any questions raised.
2. I understand that my participation is voluntary and that I am free to withdraw at any time without giving a reason and without my rights being affected in any way.
3. I understand that the researchers will hold all information and data collected securely and in confidence and that all efforts will be made to ensure that I cannot be identified as a participant in the study—I agree to take part in the above study.
4. I am over 18 years.
5. I am a staff of the University of Energy and Natural Resources, Ghana
6. **Quantitative questionnaire**
7. How often do you look at nutrition labels on food when you are purchasing?
8. Always
9. Sometimes
10. Hardly ever
11. Never—Go to Q4
12. Which items on the label do you look at? *Tick all that apply*
13. Calories
14. Total Fat
15. Saturated Fat
16. Trans fat
17. Total carbohydrate
18. Total Sugar
19. Salt
20. Other (Please specify)
21. Is there one item in particular that you are interested in? *Tick one only*
22. Calories
23. Total Fat
24. Saturated Fat
25. Trans fat
26. Total carbohydrate
27. Total Sugar
28. Salt
29. No priority
30. Which one of the following do you believe is the most important to watch in order to stay healthy? *Tick one only*
31. Calories
32. Carbohydrates
33. Sugar
34. Fat
35. Saturated fat
36. Protein
37. Alcohol
38. Salt
39. Pre-packaged food labels contain a list of ingredients. If you saw the following items listed how would you classify them? Please categorise each one as natural sugar, added/free sugar or an artificial sweetener...

*Note: Added/Free sugars are those that are added to foods during manufacturing/cooking.*

|  | **Natural Sugar** | **Added/Free Sugar** | **Artificial Sweetener** | **Don’t Know** |
| --- | --- | --- | --- | --- |
| Glucose |  |  |  |  |
| Sucrose |  |  |  |  |
| Saccharin |  |  |  |  |
| Fructose |  |  |  |  |
| Maltose |  |  |  |  |
| Honey |  |  |  |  |
| Agave nectar |  |  |  |  |
| Molasses |  |  |  |  |
| Fruit juice |  |  |  |  |
| Corn syrup |  |  |  |  |
| Aspartame |  |  |  |  |
| Invert sugar |  |  |  |  |
| Isoglucose |  |  |  |  |

1. And what about the following items—how would you classify them?

|  | **Natural Sugar** | **Added/Free Sugar** | **Don’t Know** |
| --- | --- | --- | --- |
| Sugars present in milk (Lactose) |  |  |  |
| Sugars in fresh fruit and vegetables |  |  |  |

1. Which, if any, would you actively avoid? *Tick all that apply*
2. Glucose
3. Sucrose
4. Saccharin
5. Fructose
6. Maltose
7. Honey
8. Agave nectar
9. Molasses
10. Corn syrup
11. Fruit juice
12. Aspartame
13. Invert sugar
14. Isoglucose
15. Sugars present in milk (Lactose)
16. Sugars in fresh fruit and vegetables
17. None
18. Have you heard of the World Health Organisation (WHO) recommendation for the reduction of added sugar to 5% of daily intake for additional health benefits?
19. Yes
20. No
21. The WHO published a new guideline in March 2015 recommending that sugar should make up no more than 10% of daily energy intake. It further recommended a reduction to 5% for additional health benefits. 5% is the equivalent of 9 teaspoons for men and 6 teaspoons for women.

The sugars in question are Added Sugars, i.e., sugars that are added to foods during processing or cooking and those naturally present in honey, fruit juice and syrup.

Based on current labelling how easy would it be for you to monitor and plan total sugar intake? Would you say:

1. Very easy
2. Fairly easy
3. Not very easy
4. Not easy at all
5. Don’t know
6. How do you think you would manage your sugar intake if you were trying to reduce consumption? I’m interested in the type of approach you might take.

|  |
| --- |
|  |

1. Thinking about when you go food shopping what would be the most important aid to you in understanding the sugar content of food?

|  |
| --- |
|  |

1. How helpful do you find the Traffic Light system where food elements such as Fat, Carbohydrate, Sugar and Salt are coded red, amber or green depending on the levels in the food?
2. Very helpful
3. Somewhat helpful
4. Not very helpful
5. Not helpful at all
6. Don’t know
7. How interested are you in food and nutrition? Would you say
8. Very interested
9. Interested
10. Not very interested
11. Not interested at all
12. Don’t know
13. **Demographics**
14. Gender
15. Male
16. Female
17. Age
18. 18–24
19. 25–34
20. 35–44
21. 45–54
22. 55–64
23. 65–74
24. 75+
25. Prefer not to say
26. What is the highest level of education that you have attained?
27. High school
28. College
29. Degree
30. Postgraduate
31. Prefer not to say
32. Which category of staff do you belong to at UENR
33. Senior Member Teaching
34. Senior Member Non-Teaching
35. Senior Staff
36. Junior Staff
37. Others (Please specify)……………………………
38. Are there children aged under 18 living in your household?
39. Yes
40. No
